# Gill Alterations in Adult Zebrafish Exposed to Avobenzone: Morphometric Profiling and Machine-Learning Analysis

**DOI:** 10.3390/ani16203293

**Published:** 2026-10-19

**Authors:** Sanaz Roveyshedzadeh, Anna Pinnarelli, Mehdi Forouzanfar, Khoshnaz Payandeh, Elvira Brunelli

**Affiliations:** 1 Department of Biology, Ecology and Earth Science, University of Calabria, 87036 Rende, Italy; sanazroveyshedzadeh@gmail.com (S.R.); elvira.brunelli@unical.it (E.B.); 2 Department of Mechanical, Energy and Management Engineering, University of Calabria, 87036 Rende, Italy; 3 AI Application Development Research Center, Ahv.C., Islamic Azad University, Ahvaz 61349-37333, Iran; mehdi.forouzanfar@iau.ac.ir (M.F.); khpayandeh@iau.ac.ir (K.P.)

**Keywords:** avobenzone, *Danio rerio*, gill histopathology, fish toxicology, morphometric profiling, machine learning, quantitative image analysis, ultraviolet filters

## Abstract

Gill histopathology is widely used in fish toxicology, but lesion scores and targeted measurements capture only part of the structural information in routine sections. We tested whether a morphometric profile could quantify avobenzone-related changes in adult zebrafish gills. The 40× dataset comprised gill sections from 35 adult zebrafish across five conditions represented in the image dataset: a 4-day solvent-control group and fish exposed to nominal avobenzone concentrations of 3 or 9 μg/L for 4 or 21 days. From 750 fields, quality control and automated segmentation yielded 503 accepted masks; 360 fields (72 per condition) were used in the balanced analysis. Thirty-nine descriptors quantified tissue geometry, interlamellar spaces, branch-like organisation, and thickness heterogeneity. Validation was repeated 30 times with all fields from each fish kept in the same partition. The time-matched 4-day control-versus-exposed comparison reached 89.4 ± 4.1% balanced accuracy. The 3-versus-9 μg/L comparison at 4 days reached 88.7 ± 4.6%, and 4 versus 21 days within the 9 μg/L group reached 88.5 ± 5.0%. Performance for direct five-condition classification was lower (68.4 ± 5.1%). Because 21-day control images were not included, exposure-related inference was restricted to the time-matched 4-day comparison. Morphometric profiling provides a quantitative complement to conventional histopathology while preserving individual fish identity as the grouping unit for image analysis.

## Simple Summary

Avobenzone is a common sunscreen ingredient that can reach surface waters. Fish gills are directly exposed to the surrounding water and can show structural damage after contact with pollutants. Routine histology can identify such damage, but it usually relies on lesion scores or a small number of manual measurements. Here, gill images from adult zebrafish exposed to avobenzone were analysed using 39 measurements that describe overall gill tissue structure. Images from the same fish were always kept together during analysis. A time-matched comparison at 4 days showed clear differences between control and exposed samples, and additional differences were detected between exposure concentrations and between exposed samples collected at different time points. Because the present 40× image dataset did not include a 21-day control, longer-term comparisons were interpreted only as time-point-associated differences within exposed fish. The approach does not replace expert histopathology; instead, it provides an additional quantitative description of changes already visible in the tissue. This may help make comparisons of pollutant-related gill damage more objective and reproducible.

## 1. Introduction

Fish gills are a large, highly vascularised interface between the animal and the surrounding water. In addition to gas exchange, they contribute to ion regulation, acid–base balance, and nitrogenous-waste excretion, making branchial tissue particularly responsive to waterborne contaminants [1]. Histopathology is therefore widely used in fish toxicology, but lesion scores and targeted morphometry reduce complex tissue architecture to a limited number of categorical or continuous endpoints [2]. Standardised quantitative morphometry can improve reproducibility when image selection, measurement rules, and biological replication are explicitly controlled [3]. Recent studies in zebrafish and other fish species continue to show that epithelial integrity, lamellar organisation, and interlamellar spaces are sensitive indicators of branchial stress [4]. Multi-level gill studies further support the value of structural endpoints when they are interpreted within a defined toxicological context [5].

Organic ultraviolet (UV) filters are continuously released from personal-care products into aquatic environments. Recent assessments emphasise persistent data gaps for freshwater organisms, particularly for chronic and tissue-specific effects [6]. Their occurrence in freshwater bathing areas and other receiving waters further supports the need for biologically meaningful tissue endpoints [7]. Environmental reviews have also highlighted the occurrence, discharge pathways, and ecological risks of organic UV filters [8]. Avobenzone is among the widely used organic UV filters of toxicological interest, and endocrine-disrupting potential has been discussed for this contaminant class [9]. In zebrafish, avobenzone-containing exposures have been associated with thyroid disruption and developmental effects [10]; more recent studies have extended concern to multiple UV filters across embryo-larval, juvenile, and adult life stages [11]. Life stage can influence organ-specific sensitivity [12], and subchronic exposure has been associated with endocrine and neuronal responses in juvenile and adult zebrafish [13]. Under the same adult exposure conditions used here, conventional histology, ultrastructure, morphometry, and molecular analyses reported avobenzone-associated gill alterations across complementary structural and molecular endpoints [14].

The earlier biological study established that avobenzone can alter gill structure, but it did not determine how much of the architecture visible in routine histological sections could be captured quantitatively. Conventional lesion scoring is valuable for identifying tissue damage, and manual morphometry provides direct measurements of selected structures, yet neither is designed to represent the broader combination of tissue occupancy, interlamellar spacing, branch organisation, fragmentation, and thickness heterogeneity within the same image. Digital pathology and automated image analysis offer complementary ways to standardise such measurements [15], and automated lesion identification is increasingly being explored in environmental histopathology [16]. In zebrafish toxicology, automated image analysis has also been proposed for high-throughput assessment of chemical effects [17]. Recent larval studies further illustrate how image-derived morphology can be used to support toxicological screening [18].

Quantitative analysis of fish gills is not entirely new. Fractal descriptors have been used to complement expert assessment of toxicant-related gill pathology [19], and local connected fractal analysis has captured structural changes in experimentally exposed fish gills [20]. Image-based classification has also been explored in contaminant-exposed fish [21], while neural-network approaches have been applied to hyperplastic regions in Atlantic salmon gill whole-slide images [22]. More recent work is extending automated segmentation to fish histological images [23]. These studies demonstrate that computational analysis can support gill pathology, but they do not address the specific combination considered here: a multifeature morphometric profile of adult zebrafish gill architecture evaluated across experimental conditions and within concentration- and time-point-related contrasts while preserving Fish ID as the grouping variable throughout computational validation.

The aim was not to replace conventional histopathology or to build a diagnostic classifier for avobenzone exposure. We instead asked whether structural information already present in routine gill sections could be organised into a broader morphometric profile and whether that profile reflected differences associated with experimental condition, concentration, and sampling time point. Machine learning was used only as a supporting analytical tool to evaluate these multivariate patterns. This supporting role is consistent with the broader use of learning-based methods for state estimation and fault detection in complex dynamic chemical processes [24].

This study combines a 39-descriptor profile of adult zebrafish gill architecture with fish-grouped validation and comparison with conventional structural observations obtained under the same exposure conditions. Keeping all fields from each fish in a single data partition preserved fish identity during computational validation and prevented fields from the same animal from appearing in both model-development and test partitions.

## 2. Materials and Methods

### 2.1. Fish Maintenance, Exposure Conditions, and Ethics

Adult zebrafish (*Danio rerio*), wild-type AB strain, of both sexes and 6–8 months of age, were maintained in a recirculating system at the Zebrafish Facility of the University of Bergen, Norway. Fish were acclimated for 7 days before exposure. Water parameters were maintained at 26 ± 2 °C, pH 7.5 ± 0.5, dissolved oxygen 8 ± 1 mg/L, conductivity 600 ± 100 μS/cm, and hardness 100 mg/L CaCO_3_, under a 14:10 h light:dark photoperiod. Fish were fed brine shrimp (*Artemia salina*), and no mortality was recorded during acclimation or exposure.

Avobenzone (AVO; purity ≥99%; Sigma-Aldrich, St. Louis, MO, USA) was dissolved in DMSO to prepare a working solution. Specifically, 1000 μg of AVO was dissolved in 100 μL of DMSO, and the final volume was brought to 1 L with dechlorinated tap water, yielding an AVO concentration of 1000 μg/L and a DMSO concentration of 0.01% (*v*/*v*). Appropriate aliquots of this solution were added to the aquaria to achieve nominal AVO concentrations of 3 or 9 μg/L, corresponding to the low- and high-concentration conditions, respectively. A solvent-control group was included. The 3 and 9 μg/L exposure levels were predefined by the source exposure design and were retained unchanged for the present quantitative image analysis; they were not selected or adjusted according to machine-learning performance. These concentrations were originally selected with reference to environmental occurrence: reported AVO concentrations in surface waters span approximately 0.13 ng/L to 10 μg/L, and 9 μg/L therefore represents an elevated contamination scenario within the reported range [14]. Because only these two nominal AVO levels were examined and aqueous concentrations were not analytically verified, the present study does not establish a complete concentration–response curve, a toxicity threshold, or a measured concentration–response relationship. A semi-static design with complete daily renewal of exposure medium was used, and exposure endpoints were 4 and 21 days. Because analytical verification was not performed during the exposure period, all AVO concentrations are reported as nominal. Husbandry, exposure design, and tissue processing followed the corresponding biological toxicology experiment [14]. The original supplier and purity/grade metadata for DMSO were not retained in the available source records.

The present quantitative image analysis included gill sections from 35 adult zebrafish, with seven fish represented in each of five image conditions: the 4-day solvent control, 3 μg/L AVO at 4 days, 9 μg/L AVO at 4 days, 3 μg/L AVO at 21 days, and 9 μg/L AVO at 21 days. In the source exposure experiment, each experimental group at each sampling endpoint included three replicate tanks, which were considered the experimental units. Each tank initially contained 14 fish, comprising seven males and seven females. The present image dataset was derived from one experimental repetition and therefore includes five condition-specific tanks, with one tank for each represented condition; histological images from seven fish per tank were analysed, giving 35 fish overall. Fish were randomly assigned to the experimental groups. The exact randomisation procedure was not retained in the available records, and sex was not subsequently retained as an individual sample-level variable for the histological analysis. The source exposure experiment included solvent-control fish at both the 4- and 21-day endpoints. The 21-day control condition was not represented in the present 40× image dataset because the original qualitative histological assessment found no appreciable morphological differences between the 4-day and 21-day control gills; this observation was not interpreted as quantitative equivalence between the two control conditions. No new animal exposure experiment was conducted for the present study. The histological specimens originated from the same exposure experiment reported in [14], whereas the 40× images analysed here were newly acquired from the original histological sections for the present quantitative analysis and had not been previously published or analysed as an image dataset. Accordingly, exposure-related inference was restricted to the time-matched 4-day control-versus-exposed comparison, while 4-versus-21-day comparisons were interpreted as time-point-associated differences within AVO-exposed fish. The exposure protocol from which the histological material was obtained was approved by the Norwegian Food Safety Authority (FOTS ID 31409; approval date: 27 June 2025).

### 2.2. Gill Sampling and Histological Processing

At each exposure endpoint, fish were euthanised by immersion in buffered tricaine methanesulfonate (MS-222) at 0.2 mg/mL, adjusted to pH 7–7.5. Gills were rapidly excised and processed for microscopy. Samples were fixed for 3 h in 3% glutaraldehyde (Electron Microscopy Sciences, Hatfield, PA, USA) prepared in 0.1 M phosphate buffer at pH 7.2, post-fixed in 2% osmium tetroxide (Electron Microscopy Sciences, Hatfield, PA, USA) for 2 h at 4 °C, dehydrated through a graded ethanol series, treated with propylene oxide (99% pure; Thermo Scientific, Waltham, MA, USA), and embedded in Epon–Araldite resin (Araldite 502/Embed 812; Electron Microscopy Sciences, Hatfield, PA, USA). Semi-thin sections of 1 μm physical thickness were obtained using a Leica UltraCut UCT ultramicrotome (Leica Mikrosysteme GmbH, Vienna, Austria) and stained with 1% toluidine blue (for microscopy, pure powder; Carlo Erba Reagents, Cornaredo, Italy) in 2% borate (pure powder; Merck KGaA, Darmstadt, Germany). The original supplier and/or purity/grade metadata for MS-222, phosphate buffer, and the graded ethanol series were not retained in the available source records.

### 2.3. Image Acquisition, Traceability, and Quality Control

Gill sections used for the present quantitative image analysis were imaged using a Euromex Delphi-X Observer light microscope (Euromex Microscopen B.V., Duiven, The Netherlands) equipped with an Olympus EP50 digital camera (Olympus Corporation, Hachioji, Tokyo, Japan). Images were acquired using a Plan 40× objective with a numerical aperture of 0.65 (40×/0.65; *∞*/0.17), at native image dimensions of 2592 × 1944 pixels. Pixel-to-μm calibration was performed in QuPath (version 0.6.0) using the scale bar generated on the original microscope images: a line was drawn along the full length of the scale bar and its known physical length was entered, yielding a calibrated pixel size of 0.1242 μm/pixel in both the x and y dimensions. Scale bars and textual annotations were excluded from the computational region of interest after calibration. Field acquisition and image-quality assessment followed predefined technical criteria and were performed without access to the treatment label. Eligibility was based on focus, preparation quality, staining contrast, suitable field composition, and sufficient representation of gill tissue.

The initial dataset contained 750 histological fields, with 150 fields from each of five image conditions: 4-day solvent control, 3 μg/L AVO for 4 days, 9 μg/L AVO for 4 days, 3 μg/L AVO for 21 days, and 9 μg/L AVO for 21 days. Every field retained its experimental-group label and individual Fish ID. After image quality control and visual mask acceptance, 503 fields were retained: 87 control, 104 fields from 3 μg/L AVO at 4 days, 140 from 9 μg/L AVO at 4 days, 72 from 3 μg/L AVO at 21 days, and 100 from 9 μg/L AVO at 21 days. For balanced model comparison, 72 fields were retained per group (360 fields in total). When a group contained more than 72 valid fields, fields were selected at random while preserving representation from every Fish ID. Image fields were treated as histological subsamples nested within fish, not as independent biological replicates.

### 2.4. Automated Mask Generation

**Figure 1:**
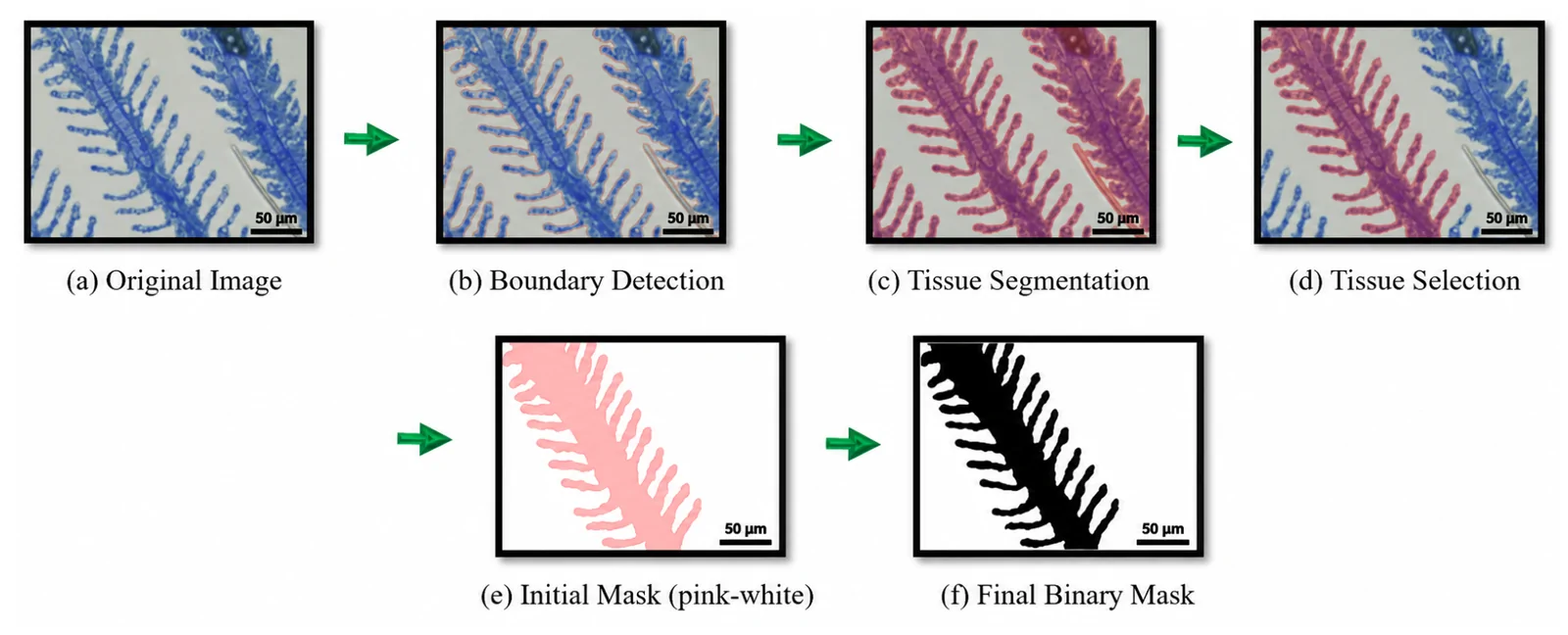
Automated mask-generation sequence. The panels show the original histological image, intermediate segmentation steps, and the final binary mask used for feature extraction. Scale bar = 50 μm in all panels; the scale-bar regions were excluded from the computational region of interest.

Images were processed using a study-specific scripted segmentation workflow developed to separate toluidine-blue-stained gill tissue from the bright background (Figure 1). General binary-image-processing principles followed standard digital image-processing practice [25]; the complete study-specific implementation and processing parameters are provided in the accompanying analysis scripts. The same segmentation settings were applied across all experimental groups. Before segmentation, fields were screened using predefined technical quality-control criteria based on focus, preparation quality, staining contrast, suitable field composition, and sufficient representation of gill tissue. Scale bars and textual annotations were excluded from the computational region of interest. During mask processing, small isolated artefacts and fragmented background objects were removed, whereas larger interlamellar spaces were deliberately preserved because they contain information on lamellar separation, narrowing, and fusion-like architectural change. Automatically generated masks were visually assessed for technical acceptability, but no manual redrawing or manual correction was performed. This clean-up and quality-control procedure was intended to limit common technical artefacts; it was not intended to detect every possible histopathological artefact.

### 2.5. Morphometric Profiling

Thirty-five primary descriptors were extracted directly from each quality-control (QC)-accepted mask, and four composite indices were derived from those descriptors, giving a 39-descriptor morphometric profile when all variables were included. The descriptors were organised into global geometry, component-level, gap, skeleton/branch, thickness, and engineered feature families. Global descriptors characterised overall tissue occupancy and shape, including area, perimeter, tissue fraction, compactness, complexity, solidity, extent, eccentricity, aspect ratio, axis lengths, and connected-component properties. Gap descriptors quantified the number, density, size, and variability of interlamellar white spaces. Skeleton- and branch-related descriptors represented branch-like organisation through skeleton length, branch points, endpoints, branch density, and approximate branch-length summaries. Thickness descriptors quantified mean, maximum, and spatial variability of mask-derived tissue thickness. These measures were interpreted as geometric descriptors and not as one-to-one surrogates for specific histological lesions. For calibrated geometric measurements, projected tissue area was calculated from the number of foreground pixels in the segmented tissue mask multiplied by the calibrated pixel area, $A=N_{\mathrm{tissue}}s_{x}s_{y}$, where $s_{x}=s_{y}=0.1242\;\mu$m/pixel. This represents the two-dimensional projected tissue area in the histological image and not the three-dimensional respiratory surface area of the gill. Mask-derived thickness descriptors were obtained from twice the Euclidean distance transform within the binary tissue mask and converted from pixels to micrometres using the same linear calibration factor. Mean, maximum, and coefficient-of-variation descriptors summarised this in-plane geometric thickness. These mask-derived thickness measurements are distinct from the physical thickness of the histological sections, which was 1 μm.

Four composite indices were included to summarise complementary architectural patterns. Each component descriptor was standardised using parameters estimated from the training data only, and the same parameters were then applied to the corresponding validation or test data. With z(·) denoting this training-based standardisation, the indices were defined as

$$\begin{array}{cc} FI & =[z(C)+z(S)-z(W)-z(GD)]/4,\\ LDI & =[z(FI)+z(SB)+z(TCV)-z(CX)]/4,\\ LHI & =[z(SL)+z(BD)+z(W)-z(C)-z(SB)]/5,\\ GADI & =[z(C)+z(S)-z(CX)+z(GCV)+z(TCV)]/5, \end{array}$$

where *C* is compactness, *S* solidity, *W* white-space fraction, GD gap density, SB short-branch fraction, TCV thickness variability, CX complexity, SL normalised skeleton length, BD branch density, and GCV gap-area variability. The signs were chosen a priori so that higher FI, LDI, and GADI values indicate greater fusion-like geometry, architectural damage, and overall disruption, whereas higher LHI values indicate greater preservation of branchial architecture. Equal weighting avoided arbitrary post hoc weights. These indices are composite geometric summaries and are not presented as independently validated biomarkers. For predictive modelling, the four composite indices were recomputed within each training partition from the primary descriptors using training-derived standardisation parameters; the same parameters were then applied to the corresponding validation or test fields. Thus, fixed index values calculated from the full dataset were not used as predictive inputs.

Supplementary Table S4 summarises the full set of 39 descriptors, their feature families, and their geometric interpretation. Exact implementation details are retained in the analysis scripts.

### 2.6. Analysis of Morphometric Profiles

For computational validation, individual fish were treated as the biological grouping units, whereas tanks were the experimental exposure units. Image fields were treated as histological subsamples nested within fish. To preserve this structure, evaluation used repeated fish-grouped validation with 30 outer repetitions; all fields from a given fish remained within a single partition. With seven histologically processed fish per experimental group, each outer split used five fish per group for model development and two fish per group for testing.

Model and feature selection were restricted to the outer-training fish and were performed using five-fold fish-grouped inner cross-validation. Scaling, feature ranking, and calculation of the four composite indices were repeated using training data only and then applied unchanged to the corresponding validation or test data. The inner validation was nested within each outer split. This two-stage fish-grouped design was used to prevent information leakage and, importantly, to ensure that fields from a test fish were never represented during model development.

Candidate classifiers comprised the following: (i) bagged decision-tree ensembles (internal output label RF_BagTrees; 200 learning cycles; maximum splits =max[2,min(25,⌊N/4⌋)], where *N* is the number of training fields; surrogate splits disabled); (ii) radial-basis-function support vector machines with one-versus-one error-correcting output codes (SVM_RBF_ECOC; BoxConstraint =1; kernel scale estimated from the training data); (iii) distance-weighted *k*-nearest neighbours (KNN_Weighted; up to seven neighbours; Euclidean distance; squared-inverse distance weighting); (iv) AdaBoostM2 decision-tree ensembles (120 learning cycles; maximum 10 splits per tree); (v) random-subspace *k*-nearest-neighbour ensembles (120 learning cycles; up to seven neighbours; approximately half of the eligible predictors sampled per learner); and (vi) linear discriminant analysis using the MATLAB (version R2024b) pseudoLinear discriminant type. The candidate feature sets comprised the 15 global descriptors, all 35 primary descriptors, the four composite indices (FI, LDI, LHI, and GADI), and the combined 39-descriptor profile. Feature-selection options comprised no feature selection or minimum-redundancy maximum-relevance (mRMR) selection [26] with requested limits of 10, 15, 25, or 30 predictors, subject to the number of eligible variables. Model and feature selection were performed separately for each comparison within the outer-training data. Five-fold fish-grouped inner cross-validation selected the configuration with the highest mean balanced accuracy, with macro-F1 used to resolve ties; imputation, scaling, and feature ranking were restricted to the corresponding training partitions. Comparison-specific summary outputs identified RF_BagTrees with mRMR Top15 for the five focused binary comparisons, whereas the direct five-condition task selected AdaBoostM2 with the global descriptor family and an mRMR Top25 request. Because the global family contains 15 eligible descriptors, all 15 global variables were retained in that five-condition configuration.

Balancing at the condition level did not force every fish to contribute the same number of image fields. To reduce unequal influence in the revised focused analyses, each training image received a weight proportional to $1/n_{i}$, where $n_{i}$ is the number of retained training images from fish *i*. Thus, the total training contribution of each fish within a class was equalised while retaining all eligible fields. For additional fish-level evaluation, predictions from held-out fields were aggregated within each fish by majority vote, yielding one prediction per fish. Condition-stratified fish-level bootstrap resampling was used to obtain 95% confidence intervals for the additional fish-level analyses, and label-permutation testing reassigned labels at the fish level while repeating feature selection, fitting, and validation. Because the current analytical cohort contains one tank per condition, these fish-level permutation results assess predictive structure within this cohort and are not interpreted as treatment-level significance across independently replicated exposure tanks.

Accuracy, balanced accuracy, and macro-F1 were used to summarise predictive performance, with balanced accuracy emphasised because it gives equal weight to class-wise sensitivity. Unless explicitly identified as an additional fish-level analysis, the main performance values are field-level estimates obtained from fields belonging exclusively to held-out fish. Balanced accuracy is reported as mean ± standard deviation (SD) across 30 repeated fish-grouped outer partitions. The SD describes split-to-split variability within this cohort and is not treated as variation among 30 independent biological replicates. The computational search space and selection rules are provided in Supplementary Table S1, and additional metrics are provided in Supplementary Table S2 where available.

Principal component analysis (PCA) was used only for descriptive visualisation of the balanced 360-field dataset. The PCA input comprised 32 primary architecture-oriented descriptors: Area, Perimeter, TissueFraction, Compactness, Complexity, Solidity, Extent, Eccentricity, AspectRatio, ConvexityDefectArea, BoundaryRoughness, ComponentCount, MeanComponentArea, StdComponentArea, LargestComponentFraction, WhiteSpaceFraction, GapCount, GapDensity, MeanGapAreaNorm, GapAreaCV, MaxGapAreaNorm, SkeletonLengthNorm, BranchPointCount, EndPointCount, BranchDensity, EndpointDensity, ApproxMeanBranchLength, ShortBranchFraction, LongBranchFraction, ThicknessMean, ThicknessMax, and ThicknessCV. MajorAxisLength, MinorAxisLength, BoundingBoxArea, and the four composite indices were not included in this descriptive PCA. Each variable was centred by its pooled mean and divided by its pooled sample standard deviation across the 360 fields; variables with zero variance would have been excluded, and none of the 32 retained variables had zero variance. This descriptive standardisation was separate from training-only preprocessing used for predictive classification.

The direct five-condition analysis was retained as a descriptive multigroup assessment of the image dataset. To avoid confounding exposure status with sampling time point, exposure-related separation was evaluated by comparing the 4-day solvent-control group with the two 4-day avobenzone-exposed groups. Additional focused comparisons were restricted to contrasts that did not mix the 4-day control with 21-day exposed fields: 3 versus 9 μg/L AVO at 4 days, 4 versus 21 days within the 9 μg/L condition, 4 versus 21 days across exposed fields, and 3 versus 9 μg/L AVO across exposed fields. Because the current 40× dataset did not include 21-day control images, pooled control-versus-all-exposed and control/low/high tasks spanning both exposure times were not used for biological inference.

## 3. Results

### 3.1. Dataset Curation and Mask Generation

Following image-quality assessment and visual mask acceptance, field retention differed among experimental conditions (Table 1). To provide equal representation of the five image conditions in the primary analysis, 72 fields per condition were retained, yielding a balanced dataset of 360 fields while preserving representation from every Fish ID.

**Figure 2:**
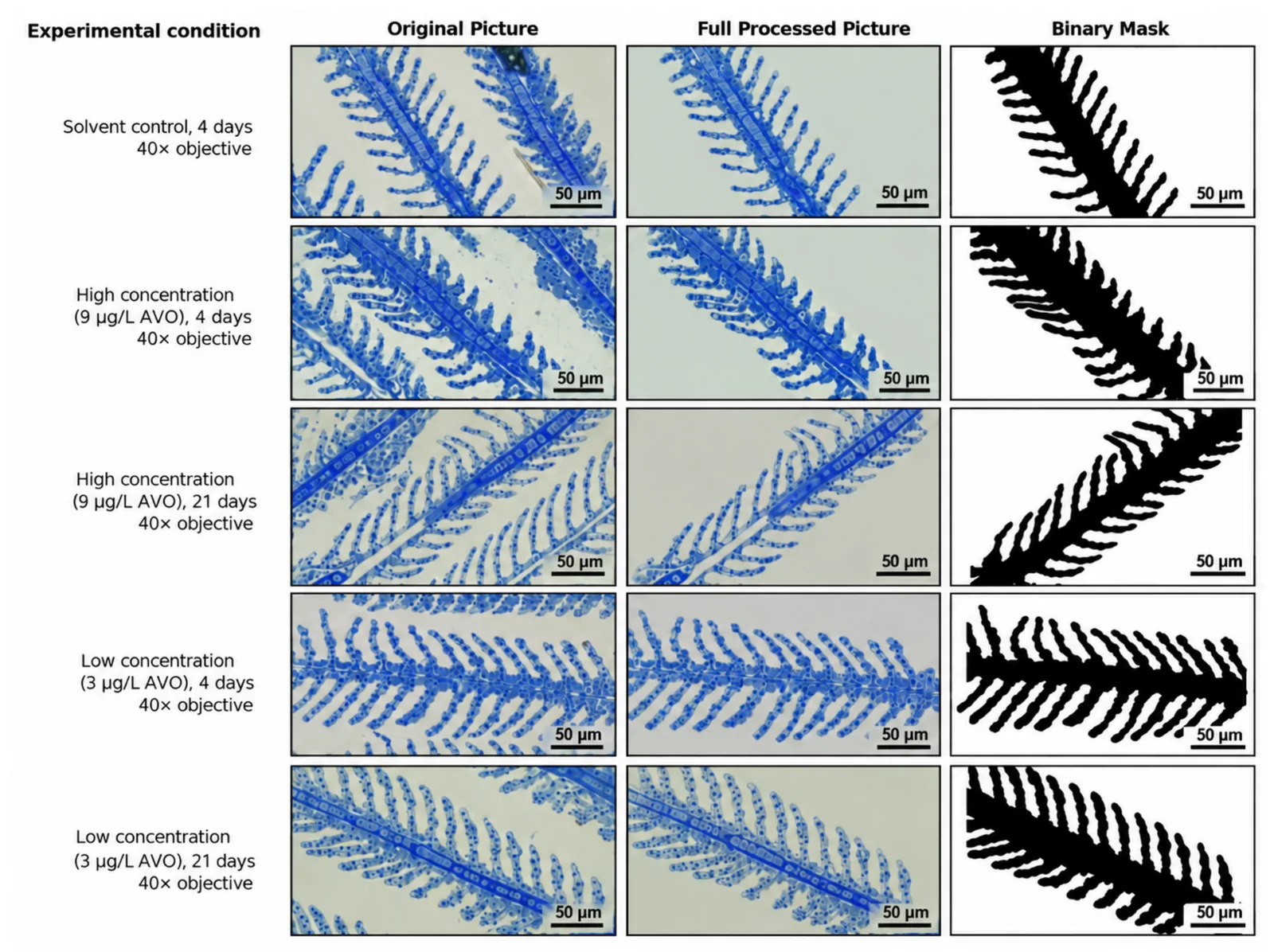
Representative segmentation outputs from the five image conditions: 4-day solvent control; nominal 9 μg/L AVO for 4 days; nominal 9 μg/L AVO for 21 days; nominal 3 μg/L AVO for 4 days; and nominal 3 μg/L AVO for 21 days. Each row shows an original gill field acquired using a 40× objective, the corresponding processed image, and the final binary mask. Larger interlamellar spaces were preserved to retain information on branchial architecture. Scale bar = 50 μm in every image panel; the scale-bar regions were excluded from the computational region of interest.

The segmentation procedure converted each accepted field into a binary representation of branchial tissue while preserving larger interlamellar spaces (Figure 1). Representative outputs from all five groups are shown in Figure 2. These masks formed the basis for subsequent feature extraction; no manual redrawing was introduced after automated segmentation.

### 3.2. Morphometric Profiling Revealed Overlapping but Shifted Structural States

The 39 descriptors formed a multivariate morphometric profile of tissue occupancy, overall shape, interlamellar spaces, branch-like organisation, fragmentation, and thickness heterogeneity. No single descriptor was expected to correspond uniquely to one lesion. Instead, the morphometric profile was designed to represent the combined architectural pattern of each field.

**Figure 3:**
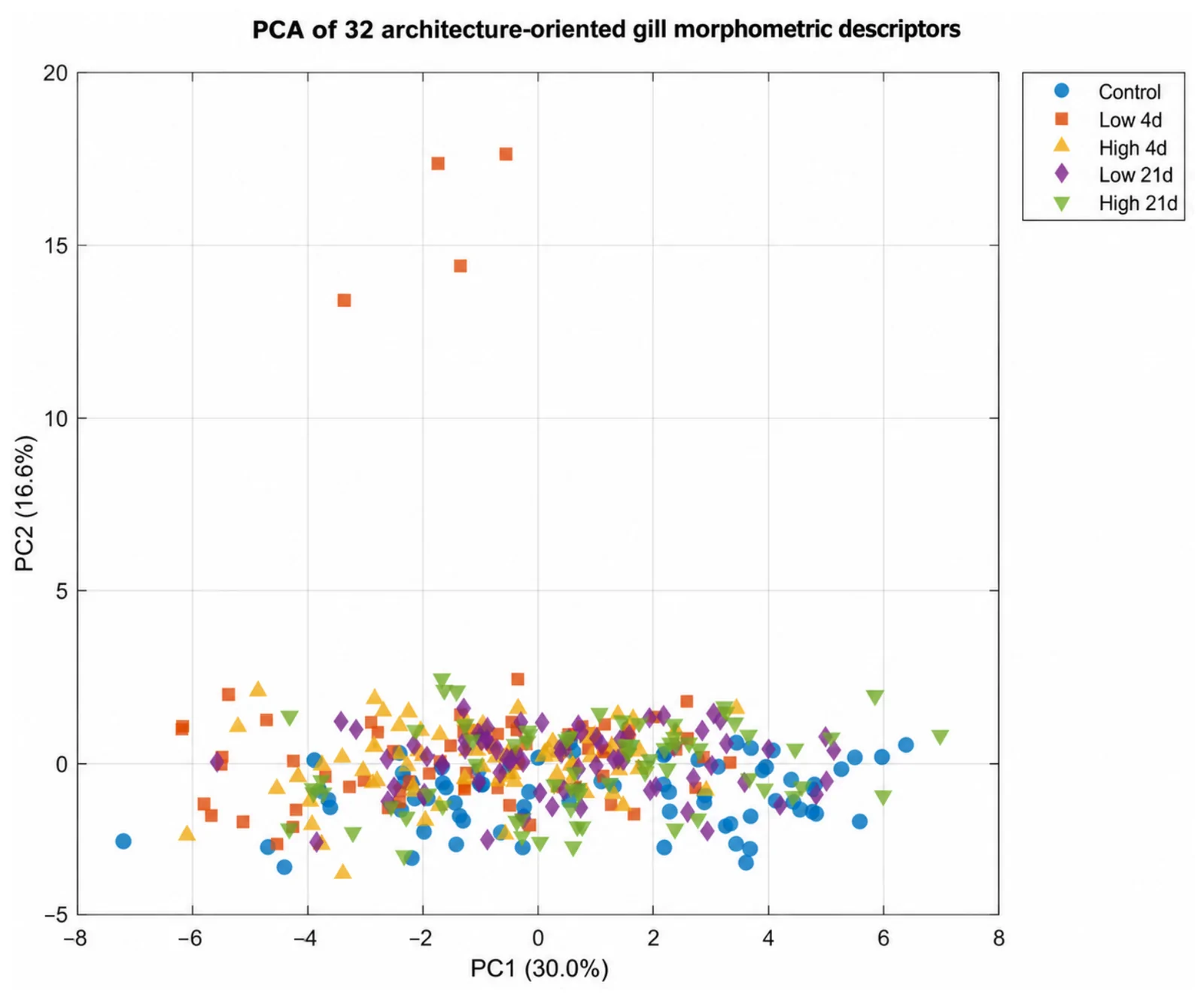
Principal-component visualisation of the 32 z-standardised architecture-oriented primary morphometric descriptors. PC1 and PC2 denote the first and second principal components, explaining 30.0% and 16.6% of the total variance, respectively. Each point represents one QC-accepted histological field. In the legend, Control denotes the 4-day solvent control; Low 4d and High 4d denote nominal 3 and 9 μg/L AVO at 4 days, respectively; and Low 21d and High 21d denote nominal 3 and 9 μg/L AVO at 21 days, respectively. The 4-day solvent-control fields show partial separation, whereas the exposed groups overlap substantially. Peripheral 3 μg/L AVO, 4-day fields were retained because they passed the same QC criteria as the remaining fields. The plot is descriptive; the 4-day control is not a time-matched reference for the 21-day exposed conditions.

PCA showed partial separation of the 4-day solvent-control fields and substantial overlap among exposed groups (Figure 3). This pattern indicates that the five image conditions occupied overlapping but shifted regions of the multivariate morphometric space. Because the image dataset did not contain 21-day control fields, the position of the 4-day control group was interpreted descriptively rather than as evidence for a time-matched exposure effect across both endpoints. Each plotted point represents one QC-accepted histological field rather than an independent fish. The peripheral 3 μg/L AVO, 4-day observations passed the same predefined QC and mask-acceptance criteria as the remaining fields and were retained. Inspection of their descriptor profiles identified particularly large normalised gap-area and mask-derived thickness values, with extreme shape descriptors in some fields. Their peripheral position therefore indicates an extreme multivariate field-level morphometric profile, but does not establish a specific histological lesion or distinguish biological heterogeneity from section orientation or segmentation effects.

### 3.3. Morphometric Profiles Showed Partial Differentiation Among the Five Experimental Groups

When the complete morphometric profile was used to distinguish the five experimental groups, balanced accuracy was 68.4 ± 5.1% across the 30 repeated fish-grouped partitions (Table 2). Model and feature settings were selected using only the corresponding training fish in each repetition; the reported value therefore reflects the complete analytical procedure rather than a single fixed classifier. Accuracy and macro-F1 are reported in Supplementary Table S2.

The 4-day solvent-control fields were more consistently distinguished within the five-condition analysis, whereas overlap occurred mainly among the exposed groups. Because no 21-day control images were included, this pattern was treated as descriptive separation among the five image conditions rather than as an estimate of exposure status independent of sampling time point. As an additional fish-level analysis, majority-vote aggregation within held-out fish yielded a five-condition balanced accuracy of 68.4%, with a condition-stratified fish-level bootstrap 95% confidence interval of 45–82% and a fish-label permutation value of p=0.01. Because the current analytical cohort contains one tank per condition, this permutation result is interpreted as evidence of predictive structure within this cohort rather than as treatment-level significance across independently replicated tanks.

### 3.4. Morphometric Profiles Differed Across Time-Matched Exposure, Concentration, and Sampling-Time Comparisons

The time-matched comparison between the 4-day solvent-control group and the two 4-day avobenzone-exposed groups reached a balanced accuracy of 89.4 ± 4.1%. Among exposed fish, the 3-versus-9 μg/L comparison at 4 days reached 88.7 ± 4.6% balanced accuracy, and 4-versus-21-day discrimination within the 9 μg/L condition reached 88.5 ± 5.0% (Table 2). Across all exposed fields, the 4-versus-21-day comparison reached 82.9 ± 5.4%, while the 3-versus-9 μg/L comparison reached 82.6 ± 5.6%. These classification values quantify the distinguishability of multivariate morphometric profiles; they do not by themselves establish the direction of individual structural changes, greater injury at the higher concentration, or progressive damage with longer exposure.

These findings indicate that the morphometric profile distinguished the time-matched exposure contrast and also captured concentration-related and time-point-associated differences in gill morphology, without requiring a pooled comparison between the 4-day control and 21-day exposed fields. The direct five-condition analysis remained useful as a descriptive assessment of the complete image dataset, but it was not used to estimate a time-independent exposure effect.

As a robustness check, analyses for which corresponding all-field outputs were available were repeated using all 503 QC-accepted fields rather than the balanced 360-field subset. Balanced accuracies differed by no more than 0.6 percentage points (Supplementary Table S3), indicating that the reported concentration, time-point, and five-condition results were not materially affected by balancing the number of image fields across conditions.

Descriptive examination of the balanced dataset further clarified which architectural dimensions contributed to the observed group separation. At 4 days, median tissue fraction was higher in the nominal 3 and 9 μg/L groups than in the solvent control (36.6% and 28.7%, respectively, versus 24.5%), whereas median white-space fraction was lower (29.6% and 30.7% versus 43.3%). Gap density was also lower in both exposed groups, describing greater tissue occupancy and reduced interlamellar white space in the sampled fields. Among the two 4-day exposed conditions, the 9 μg/L group showed a shorter normalised skeleton length than the 3 μg/L group (9.20 versus 10.91) together with a higher branch density (16.50 versus 14.04), whereas thickness variability was higher in the 3 μg/L group (0.884 versus 0.821). Within both exposed concentrations, white-space fraction and normalised skeleton length were higher at 21 days than at 4 days. The four engineered indices (FI, LDI, LHI, and GADI) were retained as complementary geometric features in the multivariate analysis. Because their predictive computation used training-partition-specific standardisation, they were not interpreted as fixed standalone group-level biomarkers; instead, their contribution was considered together with the underlying primary descriptors. Overall, the descriptive patterns did not show a consistent monotonic increase in injury with concentration or time point.

For the additional fish-level 3-versus-9 μg/L concentration analysis at 4 days, majority-vote aggregation produced a balanced accuracy of 87.5 ± 12.7%, with a fish-level bootstrap 95% confidence interval of 70–96% and a fish-label permutation value of p=0.03. These secondary fish-level results complement, rather than replace, the primary field-level results from held-out fish shown in Table 2.

## 4. Discussion

The present findings show that avobenzone-related changes in adult zebrafish gills can be described using a broader morphometric profile alongside conventional histology. The strongest separations were observed for the time-matched 4-day control-versus-exposed comparison (89.4 ± 4.1%), the 3-versus-9 μg/L comparison at 4 days, and the 4-versus-21-day comparison within the 9 μg/L condition. Discrimination among all five image conditions was more limited. This pattern indicates that exposed gills shared partially overlapping structural states rather than forming completely distinct morphological categories. The 4-day solvent-control fields also occupied a partly distinct region of the morphometric space, but the absence of 21-day control images from the current 40× dataset prevents that separation from being interpreted as a time-independent exposure effect.

This overlap is biologically plausible. Gill responses to waterborne toxicants often involve combinations of epithelial thickening, lifting, altered interlamellar spacing, shortening, deformation, and fusion-like change rather than lesions unique to a single exposure condition [27]. Recent zebrafish studies similarly report convergent structural responses to chemically distinct contaminants [28], and structural hyperplasia and architectural damage have also been described in other fish-gill exposure models [29]. In this context, the partial overlap seen in the PCA and the moderate five-group discrimination should not be interpreted as an absence of toxicological information; instead, they indicate that the sampled concentration and time-point conditions correspond to partially overlapping histological states.

Interpretation of the nominal 21-day exposure also requires consideration of avobenzone chemistry. Avobenzone can undergo photoisomerisation and photodegradation in aqueous systems, and its behaviour varies with irradiation and solution conditions including pH and temperature [30]; environmental fate and dissipation of this UV filter can also vary substantially with local conditions [31,32]. In the present semi-static design, complete daily renewal defined the dosing schedule and reintroduced the target nominal AVO level at each renewal, but it did not demonstrate that the parent-compound concentration remained constant between renewals or exclude formation of transformation products. Because neither aqueous AVO nor transformation products were analytically monitored, no tank-specific degradation rate, half-life, time-weighted aqueous concentration, or measured concentration–response relationship is inferred. The 21-day condition is therefore interpreted as repeated nominal exposure with uncertainty in the intervening chemical exposure profile.

The focused comparisons provide a clearer view of the morphometric differences. The time-matched 4-day control-versus-exposed comparison reached 89.4 ± 4.1% balanced accuracy. The 3-versus-9 μg/L comparison at 4 days reached 88.7 ± 4.6%, while 4 versus 21 days within the 9 μg/L condition reached 88.5 ± 5.0%. Broader concentration and 4-versus-21-day comparisons across all exposed fields yielded balanced accuracies of approximately 82–83%. These results show that the morphometric profiles differed across concentration and sampling-time comparisons, although the degree of separation depended on the biological contrast; the time-point comparisons are not interpreted as an isolated AVO-specific duration effect. Restricting the primary interpretation to time-matched and exposed-only contrasts avoids attributing differences between the 4-day control and 21-day exposed fields solely to avobenzone exposure.

The morphometric descriptor families were chosen to retain a direct relationship with visible branchial architecture. Approximate branch length, gap-related measures, thickness variability, compactness-related measures, and the composite architecture indices summarise interlamellar organisation, branch-like geometry, tissue compactness, and heterogeneous thickness. These properties correspond to the types of structural changes observed conventionally under the same avobenzone exposure conditions, including progressive epithelial and lamellar disruption, increased primary-filament thickness and secondary-lamellar width, and reduced secondary-lamellar length [14]. This comparison is used as a biological-consistency check; conventional morphometric, ultrastructural, and molecular endpoints were not used as inputs to the present analysis.

The approach also extends earlier efforts to quantify fish-gill morphology. Fractal analysis has been shown to complement expert assessment of toxicant-associated gill pathology [19], and local connected fractal descriptors have been applied to toxicant-exposed gills [20]. Image-based and neural-network approaches have also been explored for contaminant-exposed fish and gill histology [21]. Recent developments in environmental digital pathology further emphasise standardised lesion assessment [15] and automated analysis of fish histological images [16]. The present study differs by combining a multifeature description of adult zebrafish gill architecture with evaluation of differences across experimental conditions, concentrations, and sampling time points while preserving Fish ID throughout the analysis.

Preserving fish identity is important because multiple fields from the same animal are not independent: they reflect the same biological state and share tissue-processing, staining, and imaging characteristics. Dividing fields from the same fish between training and test data would risk information leakage and could overstate performance. The fish-grouped design used here keeps each animal entirely within one partition and restricts feature and model selection to training data. Nevertheless, this remains internal validation within a single exposure experiment and imaging protocol; an independent cohort is required to establish external generalisability. The current 40× dataset included a 4-day solvent-control condition but no 21-day control images, so pooled control-versus-exposed comparisons spanning both endpoints cannot isolate exposure from differences between sampling time points and were excluded from the primary biological interpretation. The present image-analysis cohort comprised seven histologically processed fish per represented condition (35 fish in total), with one condition-specific tank represented in this analytical dataset; therefore, independent tank replication within a condition was not available in the present image-analysis cohort. Post-QC field retention differed among groups. Although balancing was random and retained representation from every Fish ID, differential retention remains a potential source of selection bias. For the comparisons included in the all-field sensitivity analysis, using all 503 QC-accepted fields produced nearly identical balanced accuracies, supporting the robustness of the balanced primary analysis. In addition, the primary reported metrics are field-level estimates obtained from held-out fish rather than fish-level diagnostic accuracies; the additional fish-level majority-vote analyses are reported separately in Table 3. Exposure concentrations were nominal, and the absence of analytical verification means that the study cannot define a measured concentration–response relationship or toxicological threshold. Transfer to other stains, microscopes, magnifications, species, or contaminants may require recalibration. Finally, the engineered indices are geometric summaries rather than validated biomarkers.

The toxicological relevance of the study lies in adding a quantitative tissue-architecture layer to existing knowledge of UV-filter effects. Histopathological responses of fish gills have also been reported for another widely used organic UV filter, benzophenone-3 [33], and avobenzone-related endocrine effects have been documented in adult zebrafish and mixture-exposure settings [34]. The present analysis does not propose a new molecular mechanism. Instead, it provides a standardised way to describe structural information already present in gill sections, with the aim of complementing expert histopathology and supporting comparison of exposure-related branchial phenotypes.

## 5. Conclusions

A multifeature morphometric profile captured structural variation in adult zebrafish gills under the avobenzone exposure conditions examined here. The clearest differences were seen in the time-matched 4-day control-versus-exposed comparison and in selected concentration- and time-point-associated contrasts, whereas substantial overlap remained among the five image conditions. The descriptor families summarised branch organisation, interlamellar spaces, thickness heterogeneity, and fusion-like geometry and therefore provided information that can be interpreted alongside conventional histology. Because 21-day control images were not included in the present image dataset, exposure-related conclusions were restricted to the time-matched 4-day comparison; 4-versus-21-day findings within exposed fish describe time-point-associated differences and do not establish an avobenzone-specific chronic progression or exposure-by-time interaction. The approach is best viewed as a quantitative complement to expert histopathology rather than a diagnostic classifier. Independent cohorts and additional contaminants, species, imaging conditions, and time-matched long-term controls will be needed to establish broader generalisability.

## Figures and Tables

**Table 1:** Dataset composition after image quality control and visual mask acceptance.

Group	Initial Fields	Valid Masks	Balanced Analysis
Solvent control, 4 days	150	87	72
3 μg/L AVO, 4 days	150	104	72
9 μg/L AVO, 4 days	150	140	72
3 μg/L AVO, 21 days	150	72	72
9 μg/L AVO, 21 days	150	100	72
Total	750	503	360

**Table 2:** Morphometric differentiation among experimental conditions. Balanced accuracy is reported as mean ± SD across 30 repeated fish-grouped partitions. All image fields from the same fish remained within the same partition. The SD describes variability across repeated partitions and does not represent 30 independent biological replicates.

Biological Comparison	Balanced Accuracy (%)
4-day solvent control vs. 4-day exposed	89.4 ± 4.1%
3 vs. 9 μg/L AVO at 4 days	88.7 ± 4.6%
4 vs. 21 days within 9 μg/L AVO	88.5 ± 5.0%
4 vs. 21 days across exposed fields	82.9 ± 5.4%
3 vs. 9 μg/L AVO across exposed fields	82.6 ± 5.6%
Direct five-condition analysis	68.4 ± 5.1%

**Table 3:** Additional fish-level validation analyses. Held-out field predictions were aggregated by majority vote within fish. Confidence intervals were obtained by condition-stratified fish-level bootstrap resampling; permutation tests reassigned labels at fish level and repeated the analytical procedure. These results describe predictive structure within the current analytical cohort and are not treatment-level tests across independently replicated tanks.

Analysis	Fish-Level Balanced Accuracy (%)	95% CI (%)	Permutation *p*
3 vs. 9 μg/L at 4 days	87.5 ± 12.7	70–96	0.03
Direct five-condition analysis	68.4	45–82	0.01

## Data Availability

The image-derived feature matrices, QC-accepted mask-level outputs, Fish-ID mapping, analysis scripts, and part of the raw image collection are available from the corresponding author upon reasonable request, subject to institutional data-transfer and storage requirements.

## Supplementary Materials

The following supporting information can be downloaded at: https://www.mdpi.com/article/10.3390/ani16203293/s1, Table S1. Computational validation and model-selection protocol used for the morphometric analyses. Table S2. Additional performance metrics for retained morphometric analyses for which accuracy and macro-F1 outputs were available. Values are means across 30 repeated fish-grouped outer partitions. Balanced accuracy was the primary performance measure and is reported with its SD in the main manuscript. Table S3. Sensitivity analysis using all 503 QC-accepted image fields. Balanced accuracy from the primary balanced analysis (360 fields; 72 per image condition) is compared with the corresponding analysis using all QC-accepted fields. Only comparisons with corresponding all-field outputs are shown. An all-field sensitivity value was not available for the time-matched 4-day solvent-control-versus-exposed comparison. Differences are in percentage points (pp). Table S4. Overview of the 39 morphometric descriptors used in the study. Descriptions are provided at the geometric/biological inter- pretation level; exact computational implementation is retained in the analysis scripts.

## Abbreviations

The following abbreviations are used in this manuscript:

AVO	Avobenzone
CI	Confidence interval
DMSO	Dimethyl sulfoxide
ECOC	Error-correcting output codes
FI	Fusion Index
GADI	Gill Architecture Disruption Index
kNN	k-nearest neighbours
LDA	Linear discriminant analysis
LDI	Lamellar Damage Index
LHI	Lamellar Health Index
mRMR	Minimum-redundancy maximum-relevance
MS-222	Tricaine methanesulfonate
NRRP	National Recovery and Resilience Plan
PCA	Principal component analysis
QC	Quality control
RBF	Radial basis function
SD	Standard deviation
SVM	Support vector machine
UV	Ultraviolet
