# Supplementary material for: Gill Alterations in Adult Zebrafish Exposed to Avobenzone: Morphometric Profiling and Machine-Learning Analysis
Source: Animals (Basel). 2026 Oct 19;16(20):3293. doi: 10.3390/ani16203293 (PMC13700654; doi:10.3390/ani16203293)
Supplement: Supplementary file 1 [file animals-16-03293-s001.zip › animals-4577483-supplementary.pdf]

Supplementary Materials

Gill Alterations in Adult Zebrafish Exposed to Avobenzone: Morphometric Profiling and Machine-Learning Analysis

Sanaz Roveyshedzadeh, Anna Pinnarelli, Mehdi Forouzanfar, Khoshnaz Payandeh, and Elvira Brunelli

Supplementary Tables

Table S1: Computational validation and model-selection protocol used for the morphometric analyses.

Item	Specification
Outer validation	30 repeated fish-grouped holdouts. Within each original experimental group, five fish were assigned to outer training and two fish to outer testing. All fields from a fish remained in the same partition.
Inner validation	Five-fold fish-grouped cross-validation using only outer-training fish. Each inner fold held out one fish from each relevant original experimental group and used the remaining four fish per group for inner training.
Feature families	Global geometry, architecture-oriented primary descriptors, the four engineered indices, and the combined 39-descriptor morphometric profile. Exact feature membership is documented in the analysis scripts.
Feature-selection candidates	No feature selection; mRMR Top 10, Top 15, Top 25, and Top 30. When the requested top-k value was greater than or equal to the number of eligible descriptors in a feature family, the candidate was collapsed to the equivalent no-selection configuration and was not counted twice.
Classification methods	Bagged trees, AdaBoostM2 trees, weighted k-nearest neighbours, subspace k-nearest neighbours, linear discriminant analysis, and support-vector-machine configurations when applicable.
Classifier settings	Classifier-specific settings were fixed a priori and were not tuned using outer-test data. Exact implementation settings are documented in the analysis scripts.
Selection criterion	Highest mean balanced accuracy across the five inner folds; mean macro-F1 was used as the tie-breaker when required.
Preprocessing	Feature ranking, standardisation, and other training-derived transformations were fitted using inner-training data only and applied unchanged to inner-validation data. Engineered indices were recomputed within each training partition from the primary descriptors using training-derived standardisation; fixed full-dataset index values were not used for prediction. The selected configuration was then refitted on all outer-training fish before outer-test evaluation.
Biological comparisons	The direct five-condition analysis was treated as a descriptive multigroup assessment. Focused comparisons included 4-day solvent control versus 4-day exposed, low versus high concentration at 4 days, 4 versus 21 days within the high-concentration condition, 4 versus 21 days across exposed fields, and low versus high concentration across exposed fields. Pooled control-versus-exposed and control/low/high tasks spanning both endpoints were excluded from biological inference because the image dataset contained 4-day but not 21-day control fields.
Randomisation	A fixed randomisation scheme was used to generate distinct repetition-specific partitions. The implementation is retained in the analysis scripts.
Reported prediction level	Image-field level on fields from held-out fish. Predictions were not aggregated to claim fish-level diagnostic accuracy.

Table S2: Additional performance metrics for retained morphometric analyses for which accuracy and macro-F1 outputs were available. Values are means across 30 repeated fish-grouped outer partitions. Balanced accuracy was the primary performance measure and is reported with its SD in the main manuscript.

Comparison or analysis	Accuracy (%)	Macro-F1 (%)
Low vs high concentration at 4 days	94.0	90.2
4 vs 21 days within high concentration	88.5	88.5
4 vs 21 days across exposed fields	83.5	83.6
Low vs high concentration across exposed fields	83.1	83.2
Direct five-condition analysis	68.3	68.0

Table S3: Sensitivity analysis using all 503 QC-accepted image fields. Balanced accuracy from the primary balanced analysis (360 fields; 72 per image condition) is compared with the corresponding analysis using all QC-accepted fields. Only comparisons with corresponding all-field outputs are shown. An all-field sensitivity value was not available for the time-matched 4-day solvent-control-versus-exposed comparison. Differences are in percentage points (pp).

Biological comparison	Primary 360 (%)	All 503 (%)	Difference (pp)
Low vs high concentration at 4 days	88.7	88.3	-0.4
4 vs 21 days within high concentration	88.5	88.1	-0.4
4 vs 21 days across exposed fields	82.9	82.7	-0.2
Low vs high concentration across exposed fields	82.6	82.2	-0.4
Direct five-condition analysis	68.4	69.0	+0.6

Table S4: Overview of the 39 morphometric descriptors used in the study. Descriptions are provided at the geometric/biological inter-pretation level; exact computational implementation is retained in the analysis scripts.

Descriptor	Feature family	Geometric interpretation
Area	Global geometry	Foreground tissue area in the analysed mask.
Perimeter	Global geometry	Boundary length of the segmented tissue.
TissueFraction	Global geometry	Proportion of the analysed field occupied by segmented tissue.
Compactness	Global geometry	Area-perimeter based summary of how compact the segmented tissue is.
Complexity	Global geometry	Summary of shape and boundary complexity relative to overall tissue size.
Solidity	Global geometry	Segmented tissue area relative to the area of its convex hull.
Extent	Global geometry	Segmented tissue area relative to its bounding-box area.
Eccentricity	Global geometry	Elongation of the equivalent ellipse fitted to the segmented structure.
AspectRatio	Global geometry	Relative elongation based on the major and minor dimensions.
MajorAxisLength	Global geometry	Length of the major axis of the equivalent ellipse.
MinorAxisLength	Global geometry	Length of the minor axis of the equivalent ellipse.
ComponentCount	Component level	Number of connected foreground tissue components.
MeanComponentArea	Component level	Mean area of connected tissue components.
StdComponentArea	Component level	Variation in connected-component area, expressed as the standard deviation.
LargestComponentFraction	Component level	Fraction of total segmented tissue contained in the largest connected component.
ConvexityDefectArea	Component level	Area representing departures of the segmented contour from its convex hull.
BoundingBoxArea	Component level	Area of the bounding box enclosing the segmented tissue.
BoundaryRoughness	Component level	Summary of irregularity along the segmented tissue boundary.
WhiteSpaceFraction	Gap/interlamellar space	Fraction of retained non-tissue space within the analysed field.
GapCount	Gap/interlamellar space	Number of identified white-space regions retained as architectural gaps.
GapDensity	Gap/interlamellar space	Gap count normalised to the analysed spatial scale.
MeanGapAreaNorm	Gap/interlamellar space	Mean gap area normalised to the analysed spatial scale.
GapAreaCV	Gap/interlamellar space	Coefficient of variation of gap areas.
MaxGapAreaNorm	Gap/interlamellar space	Largest gap area normalised to the analysed spatial scale.
SkeletonLengthNorm	Skeleton/branch	Total skeleton length normalised to the analysed spatial scale.
BranchPointCount	Skeleton/branch	Number of branch points in the skeletonised tissue pattern.
EndPointCount	Skeleton/branch	Number of skeleton endpoints.
BranchDensity	Skeleton/branch	Branch-point abundance normalised to the analysed spatial scale.
EndpointDensity	Skeleton/branch	Endpoint abundance normalised to the analysed spatial scale.
ApproxMeanBranchLength	Skeleton/branch	Approximate mean length of branch-like skeleton segments.

Continued on next page

Table S4 – continued from previous page

Descriptor	Feature family	Geometric interpretation
ShortBranchFraction	Skeleton/branch	Fraction of branch-like segments classified as short by the fixed implementation rule.
LongBranchFraction	Skeleton/branch	Fraction of branch-like segments classified as long by the fixed implementation rule.
ThicknessMean	Thickness	Mean mask-derived tissue thickness.
ThicknessMax	Thickness	Maximum mask-derived tissue thickness.
ThicknessCV	Thickness	Coefficient of variation of mask-derived tissue thickness.
FI (Fusion Index)	Composite index	Equal-weight composite of compactness, solidity, white-space fraction, and gap density; higher values indicate more fusion-like geometry.
LDI (Lamellar Damage Index)	Composite index	Equal-weight composite incorporating FI, short-branch fraction, thickness variability, and complexity; higher values indicate greater architectural damage.
LHI (Lamellar Health Index)	Composite index	Equal-weight composite of skeleton length, branch density, white-space fraction, compactness, and short-branch fraction; higher values indicate greater preservation of branchial architecture.
GADI (Gill Architecture Disruption Index)	Composite index	Equal-weight composite of compactness, solidity, complexity, gap-area variability, and thickness variability; higher values indicate greater overall disruption.
